# Exosomes: The emerging mechanisms and potential clinical applications in dermatology

**DOI:** 10.7150/ijbs.92897

**Published:** 2024-02-25

**Authors:** Honghao Yu, Heting Feng, Hong Zeng, Yiping Wu, Qi Zhang, Jing Yu, Kai Hou, Min Wu

**Affiliations:** Department of Plastic and Cosmetic Surgery, Tongji Hospital, Tongji Medical College, Huazhong University of Science and Technology, Wuhan, 430030, China.

**Keywords:** skin, exosomes, inflammatory skin disease, autoimmune skin disease, skin regeneration

## Abstract

Skin tissue, composed of epidermis, dermis, and subcutaneous tissue, is the largest organ of the human body. It serves as a protective barrier against pathogens and physical trauma and plays a crucial role in maintaining homeostasis. Skin diseases, such as psoriasis, dermatitis, and vitiligo, are prevalent and can seriously impact the quality of patient life. Exosomes are lipid bilayer vesicles derived from multiple cells with conserved biomarkers and are important mediators of intercellular communication. Exosomes from skin cells, blood, and stem cells, are the main types of exosomes that are involved in modulating the skin microenvironment. The dysregulation of exosome occurrence and transmission, as well as alterations in their cargoes, are crucial in the complex pathogenesis of inflammatory and autoimmune skin diseases. Therefore, exosomes are promising diagnostic and therapeutic targets for skin diseases. Importantly, exogenous exosomes, derived from skin cells or stem cells, play a role in improving the skin environment and repairing damaged tissues by carrying various specific active substances and involving a variety of pathways. In the domain of clinical practice, exosomes have garnered attention as diagnostic biomarkers and prospective therapeutic agents for skin diseases, including psoriasis and vitiligo. Furthermore, clinical investigations have substantiated the regenerative efficacy of stem cell-derived exosomes in skin repair. In this review, we mainly summarize the latest studies about the mechanisms and applications of exosomes in dermatology, including psoriasis, atopic dermatitis, vitiligo, systemic lupus erythematosus, systemic sclerosis, diabetic wound healing, hypertrophic scar and keloid, and skin aging. This will provide a novel perspective of exosomes in the diagnosis and treatment of dermatosis.

## 1. Introduction

Skin, the largest organ of the human body, serves as a protective barrier against pathogens and physical trauma and plays a crucial role in maintaining homeostasis 1. It is composed of epidermis, dermis, and subcutaneous tissue, each with distinct cell types and functions. The epidermis is the outermost layer of skin and mainly consists of keratinocytes. In addition to keratinocytes and melanocytes, Langerhans cells are also found in the epidermis and are responsible for pigment synthesis and immune response respectively 2. The second layer is the dermis composed of connective tissue. Dermal fibroblasts produce collagen and elastin fibers comprising extracellular matrix (ECM), which provide strength and elasticity to the skin 3. The deepest layer of the skin is the subcutaneous tissue, which encompasses adipocytes, mesenchymal stem cells (MSCs), and blood vessels 4. Skin homeostasis depends on the complex and interconnected relationships among various skin cells, including keratinocytes, macrophages, melanocytes, fibroblasts, MSCs, and endothelial cells. Immune system disorders, inflammation, and wound can disrupt the skin barrier, consequently leading to the instability and dysfunction of skin 5. These skin-related disorders can be broadly categorized as autoimmune skin diseases, inflammatory skin diseases, and skin regeneration. These skin-related disorders are prevalent and can seriously impact the quality of patient life. Current treatment options for these skin diseases often focus on symptom management, but the limited efficacy and side effects are unsatisfactory for patients 6. Therefore, it is intriguing to understand the underlying mechanisms of skin diseases and develop targeted therapies.

Recently, exosomes have emerged as important mediators of intercellular communication 7. Exosomes are lipid bilayer vesicles derived from cells, with a diameter ranging from 30 to 100 nm 8. Regarding the distinctive biomarkers, exosomes are enriched in a group of conserved proteins, including tetraspanins (CD81, CD63, CD9), heat shock proteins (HSP70, HSP90), biogenesis-related proteins (ALIX, TSG101), and major histocompatibility complex (MHC-I, MHC-II) 9. Besides, exosomes contain multiple proteins, lipids, and nucleic acids, including mRNAs, miRNAs, lncRNAs, and circRNAs. These active cargoes in exosomes are involved in complex intercellular communication between different cells and regulate fundamental cellular processes, such as proliferation, differentiation, migration, and apoptosis 10. Therefore, exosomes can regulate the function of neighboring or distant cells to modulate inflammation, angiogenesis, fibrosis, and tissue regeneration.

In the context of skin diseases, exosomes mediate the communication of skin cells and modulate the skin microenvironment. Exosomes from skin cells, blood, and MSCs, are the main types of exosomes that are involved in modulating the skin microenvironment 11. The dysregulation of skin cell interactions and alterations in exosome cargoes are the participants in the complex pathogenesis of chronic inflammatory and autoimmune skin diseases 12. Therefore, exosomes are promising diagnostic and therapeutic targets for skin diseases. Circulating exosomes of patients with skin diseases have specific nucleic acid protein expression profiles for the diagnosis of skin disease. The cargoes in circulating exosomes can be transferred to skin cells along with blood flow to promote the development of skin diseases 13. For example, circulating exosomal miRNAs may serve as auxiliary indicative biomarkers for the diagnosis and progression of vitiligo 14. In addition, exogenous exosomes, especially MSC-exos, possess huge potential in skin disease treatment and skin regeneration 5. Exogenous exosomes possess the distinctive properties of transferring biologically active molecules and modulating cellular responses, contributing to the restoration of cellular function and the immune microenvironment in skin diseases 15. Compared with traditional dermatological therapy, exosome as a cell-free therapy offers several advantages, including targeted delivery, low toxicity, facilitated tissue repair, personalized treatment, and multifunctionality 16. These advantages endow exosome-based therapy promising frontier for more effective, safe, and personalized treatment options for skin diseases.

This review aims to provide a comprehensive summary of the most recent research findings regarding the mechanisms and applications of exosomes of dermatology, including psoriasis, atopic dermatitis (AD), vitiligo, systemic lupus erythematosus (SLE), systemic sclerosis (SSc), diabetic wound healing, hypertrophic scar and keloid, and skin aging. We hope this will provide further insights into the mechanisms and applications of exosomes in dermatology and evidence supporting the utilizing exosomes as novel diagnostic biomarkers and therapeutic agents in skin disease. To achieve goals, we conducted a literature search on Pubmed using the keywords “exosomes” and the 8 related fields within the past 5 years. The identified studies involved in cell, animal experiments, or clinical trials, especially the original articles were included according to relevance to the topic.

## 2. Exosomes in inflammatory skin disease

### 2.1 Psoriasis

Psoriasis is a chronic inflammatory skin disorder characterized by erythematous plaques, scaling, and pruritus. The main pathological manifestations are the abnormal proliferation of keratinocytes and immune dysregulation 17. Immune cells are infiltrated in psoriatic lesions, including macrophages, T cells, dendritic cells (DCs), and neutrophils. The interaction between immune cells and keratinocytes, facilitated by exosomes and inflammatory cytokines, promotes the development of psoriasiform skin inflammation 18.

#### 2.1.1 Exosomes in psoriasis pathogenesis

**Figure 1:**
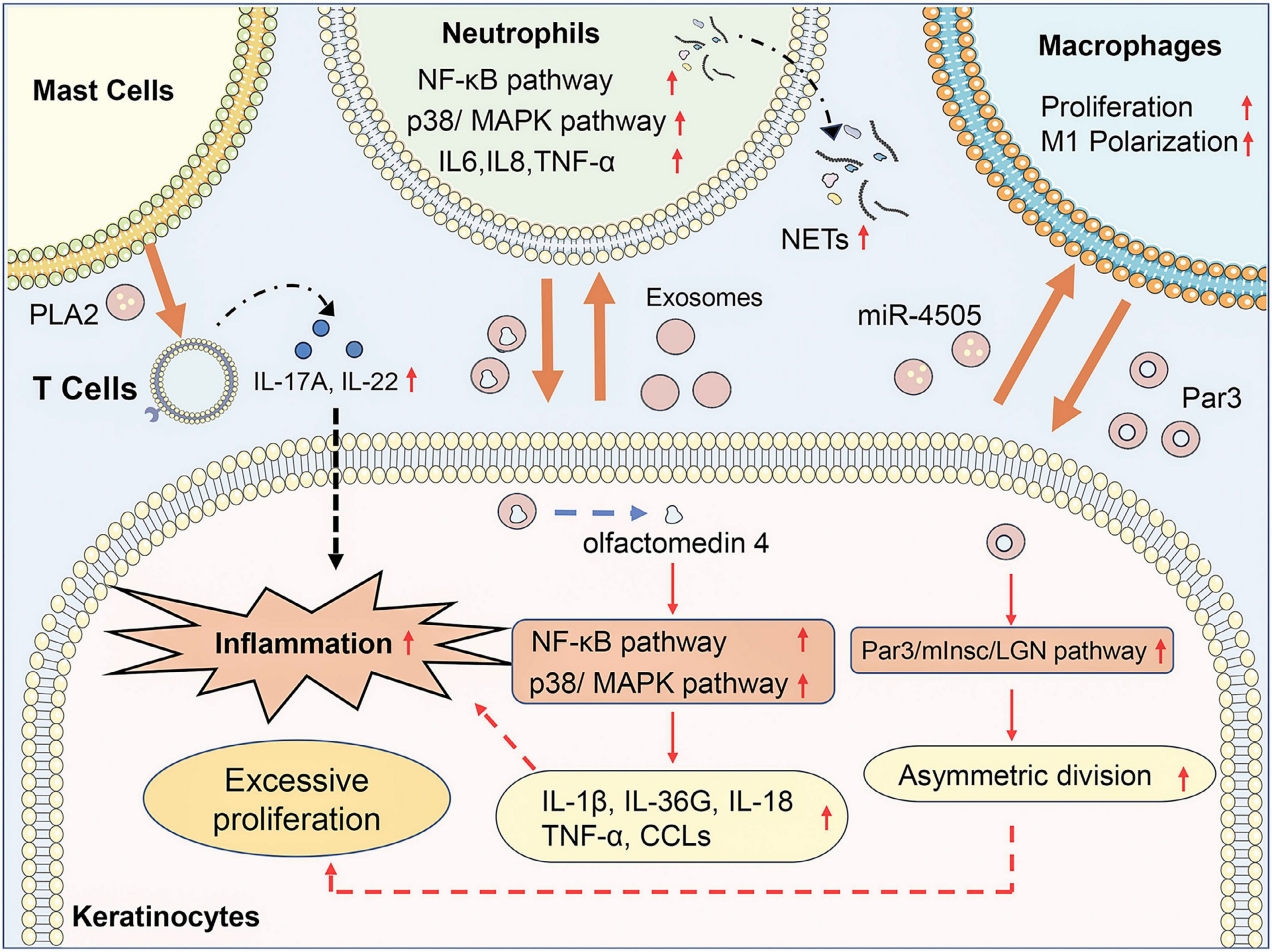
** Exosomes in psoriasis pathogenesis.** In psoriasis, keratinocyte exosomes promote neutrophil extracellular trap (NET) formation and pro-inflammatory cytokine expression in neutrophils via activating the NF-κB and p38/MAPK pathway and promote their proliferation and M1 polarization of macrophages via transferring miR-4505. Neutrophil exosomes containing olfactomedin 4 activate NF-κB and MAPK pathway in keratinocytes to promote expression of IL-1β, IL-36G, IL-18, TNF-α, and C-X-C motif chemokine ligands (CCLs). Macrophage exosomes manipulated the asymmetric division of keratinocytes via activating the Par3/mInsc/LGN signaling pathway. Mast cell-released exosomes transferred cytoplasmic PLA2 to CD1a-reactive psoriatic T cells inducing the production of IL-22 and IL-17A in psoriasis patients. In conclusion, exosome-mediated communication between activated keratinocytes and infiltrated immune cells contributes to the progression of psoriasis.

Exosome-mediated communication between activated keratinocytes and infiltrated immune cells contributes to the progression of psoriasis **(Figure 1)**. Jiang et al. found that psoriasis-like keratinocytes, which were induced by IL-17A, IL-22, and TNF-α, promoted neutrophil extracellular trap (NET) formation and pro-inflammatory cytokine expression in neutrophils via activating the NF-κB and p38 MAPK pathway 19. Besides, subcutaneously injection of exosomes from imiquimod (IMQ)-treated epidermis exacerbates the psoriasis-related inflammation and neutrophils infiltration in IMQ-induced psoriasis mouse models. Sun et al. demonstrated that keratinocytes from psoriasis patients had a lower level of vitamin D receptor (VDR) expression, and exosomes from VDR-deficient keratinocytes transferred miR-4505 to macrophages, promoting their proliferation and M1 polarization 20. It indicated that keratinocyte exosomes affected the epidermal microenvironment of psoriasis via regulating the function of neutrophils and macrophages. Moreover, immune cells could also interact with other immune cells and keratinocytes through exosomes, which modulated immune responses and led to the progression of psoriasis. Neutrophils from patients with generalized pustular psoriasis secreted more exosomes than controls and induced higher expressions of inflammatory genes, including IL-36G, TNF-α, IL-1β, IL-18, and C-X-C motif chemokine ligands (CCLs) in keratinocytes via activating MAPK and NF-κB signaling pathways 21. Proteomic profiles of exosomes revealed that olfactomedin 4 might mediate the inflammatory responses of generalized pustular psoriasis. Macrophage-derived exosomes manipulated the asymmetric division of keratinocytes to produce a large number of transit-amplifying cells in psoriasis via activating the Par3/mInsc/LGN signaling pathway 22. IFN-α-treated mast cells released exosomes containing cytoplasmic PLA2, which transferred to CD1a-reactive psoriatic T cells and led to the generation of neo-lipid antigens 23. The neolipid antigens were subsequently recognized by lipid-specific CD1a-reactive T cells and induced the production of IL-22 and IL-17A in psoriasis patients.

Circulating exosomes from patients with psoriasis have specific gene expression profiles and phospholipid profiles, which are correlated with the severity of psoriasis. IL-23 and TNF-α mRNA expression levels in circulating exosomes of psoriasis patients were significantly higher than those in healthy controls, and circulating exosomal IL-23 level was significantly positively correlated with severity index score 24. Moreover, circulating exosomal IL-17A level was significantly higher in patients with moderate-to-severe psoriasis compared with those with mild psoriasis 25. Chen et al. performed miRNA sequencing for serum exosome of psoriasis vulgaris patients and healthy controls. They identified 246 differentially expressed miRNAs between the two groups and found that the predicted target genes of differentially expressed miRNAs were enriched in metabolic processes 26. Paolino et al. demonstrated that the plasma of psoriatic patients had a higher concentration of exosomes in comparison to that of healthy controls 27. The phospholipid profile of exosomes showed that phosphatidylcholine (PC), phosphatidylethanolamine (PE), phosphatidylglycerol, and lysoPC levels were increased in psoriatic patients. Besides, the ustekinumab therapy on psoriatic patients reverted the PE and PC lipid composition of circulating exosomes toward that of healthy control. PE and PC levels of circulating exosomes could be used in the diagnosis and therapeutic effect prediction of psoriasis.

#### 2.1.2 Exosomes in psoriasis application

Exosomes from stem cells, including epidermal stem cells (ESCs), human umbilical cord MSCs (hucMSCs), and umbilical cord blood mononuclear cells (UCB-MNCs), are proven to alleviate psoriasis through immune regulation. Topical application of ESC-exos relieved IMQ-induced psoriasis in mice via reducing IL-17 and terminal complement activation complex C5b-9 in the skin 28. Topically applied exosomes are mainly localized to the stratum corneum of human skin. Besides, subcutaneous injection of hucMSC-exos significantly reduced the IL-23, IL-17, and CCL20 expression of keratinocytes and inhibited the maturation and activation of DCs in IMQ-induced psoriasis mice via down-regulating STAT3 phosphorylation 29. In addition, Rodrigues et al. found that UCB-MNC-exos promoted the differentiation of anti-inflammatory M2 macrophages and inhibited the proliferation and cytokine production of activated T cells. UCB-MNC-exos also reduced psoriasis-related inflammation in vitro 3D Model and an IMQ-induced psoriasis mouse model 30.

### 2.2 Atopic Dermatitis

AD is a chronic inflammatory skin condition characterized by intense itching, redness, and dryness of the skin 31. The pathogenesis of AD is thought to be related to impaired skin barrier function, immune dysregulation, and skin microbiota dysbiosis 32.

#### 2.2.1 Exosomes in AD pathogenesis

Skin microorganisms have been proven to affect AD-related inflammation by secreting exosomes or changing the contents of skin cell exosomes. *S. aureus* and *C. albicans* frequently colonized the atopic skin. Extracellular vesicles (EVs) from* S. aureus* promoted the secretion of pro-inflammatory cytokines in human dermal fibroblasts (HDFs), and induced AD-like inflammation in *SKH-HR1* mice via thickening epidermal increasing mast cell and eosinophil infiltration 33. Besides, Kobiela et al. reported that *S. aureus* enhanced filaggrin loading into the keratinocyte exosome via a Toll-like receptor 2 (TLR2)‐mediated mechanism. The filaggrin removal system protected the keratinocytes from keratinocyte premature death and epidermal barrier dysfunction and helped safeguard bacterial growth 34. Kobiela et al. also reported that AD cytokines and *C. albicans* collaboratively changed the surface glycosylation pattern of keratinocyte exosomes to increase their interaction with DCs via inhibitory Siglec receptors 35. In patients with AD, mast cells mediated immediate hypersensitivity and led to the release of various inflammatory mediators, such as histamine, cytokines, and chemokines. Kwon et al. reported that exosomes derived from dinitrochlorobenzene (DNCB)-treated *Nc/Nga* mouse skin mast cells enhanced the invasion and autophagy of HaCaT cells and dermal fibroblast cells by up-regulating HDAC6/CXCL13/SIRT1 axis 36. Furthermore, circulating exosomes of pediatric AD patients significantly increased the apoptosis rate and the expression levels of the pro-inflammatory cytokines in keratinocytes, including K6, K10, TSLP, and IL-33 37. Meng et al. reported that over a hundred transfer RNA-derived fragments (tRFs) from plasma exosomes were differentially expressed in pediatric patients with AD than in healthy controls. Among them, tRF-28-QSZ34KRQ590K was predicted to be involved in multiple functions and pathways associated with AD and exhibited significance in the receiver operating characteristic (ROC) curve 38. Thus, exosomes are involved in the pathogenesis of AD and might be potential biomarkers for diagnosis and targeted treatment of AD.

#### 2.2.2 Exosomes in AD application

Adipose-derived stem cell (ADSC)-exos are proven to effectively suppress inflammatory responses and promote skin barrier repair in patients with AD, thereby offering a potential therapeutic approach for treating AD. Cho et al. reported that intravenous or subcutaneous injection of ADSC-Exos ameliorated AD symptoms in *NC/Nga* mice treated with house dust mite via decreasing serum IgE levels, immune cell infiltration, and the IL-4, IL-23, IL31, and TNF-α expression 39. Besides, subcutaneous injection of ADSC-exos promoted epidermal barrier repair in an oxazolone-induced AD mouse model via enhancing stratum corneum hydration decreasing the levels of inflammatory cytokines, such as IL-4, IL-5, IL-13, IL-17, IFN-γ, TNF-α, and TSLP, and stimulating the production of epidermal ceramide 40. Meanwhile, deep RNA sequencing analysis of skin lesions demonstrated that ADSC-exos normalized the expression of genes altered during AD pathogenesis, which involved skin barrier, lipid metabolism, cell cycle, and inflammatory response. Shi et al.reported that miR-147a-overexpressing ADSC-exos inhibited inflammatory response, cell apoptosis, and angiogenesis in the DNCB-induced AD mouse model via targeting VEGF-A and MEF2A-TSLP axis 41. In addition, exosomes based on skin component cells or other stem cells, such as dermal fibroblasts and iPSC-derived MSCs (iMSCs), possess outstanding advantages in modulating inflammation and promoting tissue regeneration and repair and are expected to be candidates for AD therapy. Yoo et al. reported that exosomes derived from dermal fibroblasts restored expression levels of skin permeability barrier maintenance biomarkers in DNCB-treated keratinocytes, such as FLG, LOR, IVL, and HAS1, thus increasing the recovery rate of skin damage in AD 42. Yoon et al.reported that topical application of exosomes derived from IFN-γ-primed iMSCs significantly improved clinical and histological outcomes of Aspergillus fumigatus-induced AD mouse model, including epidermal thickness, inflammatory cell infiltration, transepidermal water loss (TEWL) level and serum IgE level 43.

## 3. Exosomes in autoimmune skin disease

### 3.1 Vitiligo

Vitiligo is a chronic skin disorder characterized by the loss of melanocytes, resulting in depigmented patches on the skin. Genetic predisposition, oxidative stress, and immune dysregulation are thought to contribute to the destruction of melanocytes 44. Treatment options for vitiligo include topical corticosteroids, calcineurin inhibitors, phototherapy, and surgical interventions 45. However, the effectiveness of these treatments varies, and there is still a need for more targeted and personalized therapies for vitiligo.

#### 3.1.1 Exosomes in vitiligo pathogenesis

Circulating exosomal miRNAs contribute to melanocyte dysregulation in vitiligo and are associated with the progression of vitiligo. For instance, circulating exosomal miR-493-3p was significantly increased in segmental vitiligo (SV) patients. MiR-493-3p-overexpressing keratinocyte induced melanocyte apoptosis and decreased melanin synthesis through inhibiting HNRNPU expression and increasing dopamine secretion 46. Circulating exosomes from vitiligo patients, containing miR-21-5p, inhibited melanin content, tyrosinase activity, and melanogenesis-related protein expression in melanocytes via inhibiting SATB1 expression 47. Moreover, Luo et al. reported that exosomal hsa-miR-487b-3p in serum was significantly lower in vitiligo patients and positively correlated with the area of lesions in progressive vitiligo patients 14. Keratinocytes are the main cell type in the epidermis and play an important role in maintaining melanocyte homeostasis. Zhao et al. demonstrated that miR-200c expression was significantly declined in keratinocyte exosomes from vitiligo lesions than that from healthy skin. And miR‐200c from keratinocyte exosomes up-regulated the expression of melanogenesis‐related genes in melanocytes via inhibiting SOX1 and activating β‐catenin 48. Overall, by carrying specific miRNAs, circulating exosomes and keratinocyte exosomes played crucial roles in the pathogenesis of vitiligo **(Table 1)**.

### 3.2 Systemic lupus erythematosus

SLE is a complex autoimmune disease characterized by the production of autoantibodies and immune system dysregulation 49. Dysregulation of the immune system leads to the production of autoantibodies that target various organs and tissues, resulting in inflammation and tissue damage 50. Ongoing research aims to develop targeted therapies that modulate the immune response and improve outcomes for SLE patients.

#### 3.2.1 Exosomes in SLE pathogenesis

SLE serum exosomes are immunologically active and can be used as a novel biomarker to predict SLE disease activity **(Table 2)**. Serum exosome levels are significantly higher in SLE patients than in healthy controls and were positively correlated with SLE disease activity. Treatment of SLE serum exosomes up-regulated the expression of IL-1β, IFN-α, TNF-α, and IL-6 in healthy peripheral blood mononuclear cells (PBMCs) in a TLR-dependent manner 51. Song et al. performed RNA-sequencing and proteomic analysis and revealed that the differently expressed exosomal miRNAs and proteins mainly enriched in pathways associated with inflammation and autophagy 52. Li et al. found that exosomal miR-21 and miR-155 were elevated in the serum of SLE patients compared to healthy controls, with even higher levels in SLE patients with lupus nephritis (LN) 53. Salvi et al. indicated that plasma exosomes from SLE patients activated the secretion of IFN-α in human plasmacytoid DCs 54. Besides, exosome-delivered miRNAs containing an IFN induction motif promoted activation, maturation, and survival of human plasmacytoid DCs in SLE patients via stimulating TLR7 expression.

T-cell exosomes from SLE patients could induce tissue inflammation via transferring specific proteins **(Table 2)**. The higher expression of eosinophil cationic protein (ECP) in T cell-derived exosomes from SLE patients compared to healthy controls. Exosomes derived from T cells of Lck-ECP-transgenic mice increased autoantibody levels, tissue IFN-γ levels, and induced hepatitis, nephritis, and arthritis in recipient mice 55. Proteomics analysis identified that bactericidal/permeability-increasing protein (BPI) was highly expressed in T cell exosomes from SLE patients compared to that from healthy controls 56. Exosomes from BPI-overexpressing T cells stimulated IL-1β production of macrophages and induced liver, kidney, and joint inflammatory responses in recipient mice. These indicated that ECP and BPI in the T cell-derived exosomes might be a key pathogenic factor for SLE-related tissue inflammation. Furthermore, T cell exosome levels were positively correlated with the activation of myeloid cells and lymphocytes in SLE, which might be mediated by type I IFN signaling 57.

#### 3.2.2 Exosomes in SLE application

Bone marrow mesenchymal stem cells (BMSCs)-exos can promote M2 macrophage polarization and decrease T cell infiltration, leading to the alleviation of clinical symptoms associated with SLE. BMSC-exos increased the radio of M2-like macrophages and reduced the T cell infiltration in the kidney to alleviate SLE nephritis in MRL/lpr mice via transferring miR-16 and miR-21 58. Dou et al. suggested that tsRNA-21109 was down-regulated in SLE patients compared to healthy control, and BMSC exosomal tsRNA-21109 alleviated SLE by inhibiting macrophage M1 polarization 59. In addition to inducing M2 macrophage polarization like BMSC-exos, hucMSC-exos also regulated the balance of the T cell subset and the activation of B cells in SLE. It was reported that intravenous injection of hucMSC-exos alleviated nephritis, liver, and lung injuries of MRL/lpr mice via increasing the radio of M2 macrophages and the expansion of Tregs 60. The ratio between Th17 and Treg cell subsets was increased in SLE patients compared to healthy controls. HucMSC-exos restored the balance of Th17/Treg and inhibited inflammatory cytokine secretion of PBMCs from SLE patients via delivering miR-19b and inhibiting KLF13 expression 61. Additionally, Zhao et al. detected the effect of hucMSC-exos on B cells selected from PBMC of SLE patients, showing that hucMSC-exos promoted B cell apoptosis, prevented B cell overactivation, and reduced IL-16, IL-10, and TNF-α expression of B cells via transferring miR-155 and activating ERK pathway 62. In conclusion, hucMSC-exos inhibited the overactivation of B cells in SLE patients via the miR-155/SHIP-1/ERK axis. Among the drugs currently used to treat SLE, immunosuppressants are often limited due to their risk of side effects. Compared to traditional therapies, MSC-exos have the potential for targeted delivery with minimal side effects and can regulate the immune response, reduce inflammation, and promote tissue repair to address the underlying pathogenesis of SLE 63. In addition, MSC-exos are easier to preserve and manage with low immunogenicity and easily cross the blood-brain barrier compared to stem cell therapy.

### 3.3 Systemic sclerosis

SSc, also known as scleroderma, is a chronic autoimmune disease characterized by fibrosis and vascular abnormalities 64. Immune dysregulation leads to the activation of fibroblasts and excessive production of collagen, resulting in tissue fibrosis and vascular damage. Treatment options consist of immunosuppressants, vasodilators, and medications to manage specific organ involvement 65. The development of targeted therapies that modulate the immune response and prevent fibrosis in SSc is a current research concern.

#### 3.3.1 Exosomes in SSc pathogenesis

Fibroblasts are key components of the dermis and their overactivation is a characteristic feature of SSc. Activated fibroblasts lead to the excessive production and deposition of ECM components, which contributes to the characteristic skin thickening and organ fibrosis in SSc patients. Bhandari et al. found that exosomes from human SSc dermal fibroblasts increased pro-inflammatory and pro-fibrotic cytokine secretion of macrophages compared with those incubated with healthy control fibroblasts 66. Besides, co-culture with macrophages pretreated with SSc exosomes promoted the collagen and fibronectin production of fibroblasts. This indicated that macrophages and fibroblasts were cooperative mediators of fibrosis in SSc. In addition, the expressions of exosomal biomarkers CD63, CD81, and CD9 were significantly increased in SSc dermal fibroblasts 67. Exosomes derived from SSc dermal fibroblasts stimulated the expression of type I collagen in normal fibroblasts. Interestingly, SSc patients with vascular involvement had reduced levels of serum exosomes, while exosomes derived from serum accelerated skin ulcer healing in mice. These findings suggested that vascular abnormalities in SSc might disrupt the transfer of exosomes from skin tissue to the bloodstream, leading to delayed wound healing. Exosomes from neutrophils induced inflammation, vasculopathy, and fibrosis in the pathological process of SSc. Li et al. found that the differently expressed miRNAs and lncRNAs between the neutrophil exosomes from diffuse cutaneous systemic sclerosis (dSSc) patients and those from healthy controls were enriched in Wnt, AMPK, IL-23, and NOTCH signaling pathways 68. Besides, dSSc neutrophil exosome treatment up-regulated the fibrosis-related gene expression of human dermal microvascular endothelial cells (HDMECs) and fibroblasts. Moreover, neutrophil exosomes from SSc patients suppressed the proliferation and migration of HDMECs via up-regulating S100A8/A9 69. Circulating exosomes also participated in the fibrosis prosses in SSc. Wermuth et al. demonstrated that 6 profibrotic miRNAs were increased and 10 antifibrotic miRNAs were decreased in SSc serum exosomes 70. Serum exosomes isolated from SSc patients caused dose-dependent stimulation of fibrosis-related genes and myofibroblast activation in HDFs in vitro.

#### 3.3.2 Exosomes in SSc application

Recently, multiple MSC-exos can reduce fibrosis of fibroblasts and regulate the function of immune cells to ameliorate SSc. HucMSC-exos alleviated skin fibrosis, suppressed the epithelial-mesenchymal transition (EMT) process, and promoted M1 polarization of macrophages in BLM-treated mice 71. In another research, hucMSC-exos reduced dermal thickness, down-regulated fibrosis-related gene expression, and inhibited myofibroblast activation in BLM-treated mice via suppressing TGF-β/Smad signaling pathway 72. In addition, MSC-exos enriched anti-fibrotic and anti-inflammatory miRNAs, which were potential for the treatment of SSc. BMSC-exos attenuated the fibrosis of BLM-treated mice via transferring miR-214 to inhibit the IL-33/ST2 axis 73. Besides, miR-196b-5p from BMSC-exos significantly inhibited BLM-induced dermal fibrosis in mice via inhibiting collagen production, myofibroblast activation, and infiltration of macrophages and CD3+T cells in lesional skin 74. Rozier et al. reported that ADSC-exos attenuated clinical symptoms and down-regulated the expression of several pro-fibrotic, remodeling, and anti-apoptotic factors in HOCl-induced mouse SSc model via delivering miR-29a-3p 75. These findings highlight the potential of MSC-exos and their cargoes as promising therapeutic strategies for SSc.

## 4. Exosomes in skin regeneration

### 4.1 Diabetic wound healing

**Figure 2:**
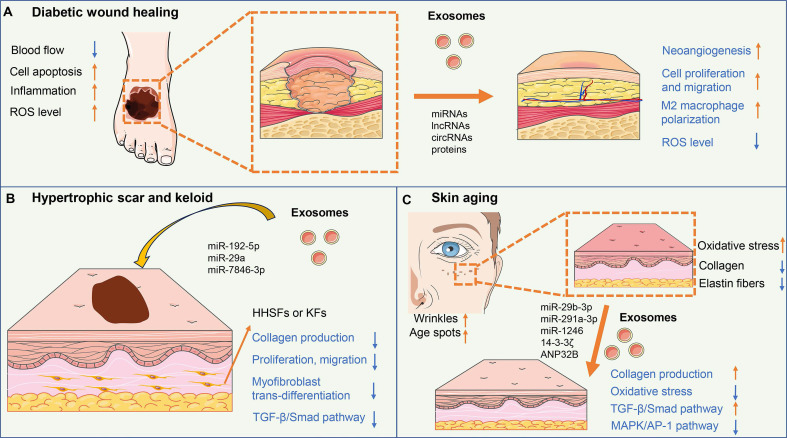
** The mechanisms of exosomes in skin regeneration.** (A) Exosomes could improve angiogenesis, cell proliferation and migration, and M2 macrophage polarization, and reduce ROS level, thus accelerating diabetic wound healing. (B) ADSC-exos containing miR-29a, miR-192-5p, and miR-7846-3p inhibit collagen production, proliferation, migration, and myofibroblast trans-differentiation of fibroblasts via targeting TGF-β/Smad pathway, resulting in reduced excessive fibrosis and scar formation. (C) Exosomes could up-regulate collagen synthesis and reduced oxidative stress of fibroblasts via activating the TGF-β/Smad pathway and inhibiting the MAPK/AP-1 pathway, thus enhancing skin elasticity and easing the wrinkles for anti-aging.

Diabetic wounds are a common complication of diabetes mellitus and are characterized by impaired wound healing and increased risk of infection 76. High blood sugar levels in diabetes can lead to damage to blood vessels, resulting in reduced blood flow to the wound site and delayed wound healing. Additionally, immune dysfunction in diabetes impairs the ability to fight infection and causes persistent inflammation of the wound area. The clinical treatment for diabetic wounds mainly includes wound debridement, infection control, and the use of dressings, growth factors, and negative pressure wound therapy 77** (Figure 2A)**.

#### 4.1.1 Exosomes in diabetic wound pathogenesis

During the process of diabetic wound healing, exosomes mediate the intercellular interaction among various skin cells, including keratinocytes, fibroblasts, macrophages, endothelial cells, and ESCs. Macrophage-derived exosomes accelerated diabetic wound healing by inducing angiogenesis, enhancing cell bioactivity, and inhibiting inflammatory cytokines 78. Lean adipose tissue macrophage-derived exosomes promoted M2 macrophage polarization to improve diabetic wound healing via transferring miR-222-3p to inhibit Bim expression in mice 79. ESC-exos induced M2 macrophage polarization to promote diabetic wound healing via miR-203a-3p/SOCS3-mediated JAK2/STAT3 signaling activation 80. ESC-exos accelerated diabetic wound healing via promoting cell proliferation, angiogenesis, and M2 macrophage polarization. ESC-exos regulated the phosphatidylinositol-3 kinase/protein kinase B and TGF-β signaling pathways via delivering miRNAs 81. Endothelial progenitor cell-derived exosomes (EPC-exos) promoted proliferation and migration while inhibiting apoptosis of HaCaTs challenged by HG via miR-182-5p/ PPARG axis, thus accelerating diabetic wound healing 82. EPC-exos promoted angiogenesis in diabetic wound healing via delivering miRNA-221-3p 83. Exosomes derived from autologous dermal fibroblasts improved angiogenesis and epithelial regeneration in diabetic wounds via activating the AKT/β-catenin pathway 84. Exosomes from human keratinocytes enhanced the phagocytosis and pro-healing function of macrophages to promote diabetic wound healing via MALAT1/miR-1914-3p/FGE8 axis 85.

Endogenous circulating exosomes are involved in the progression of diabetic wounds. MiR-20b-5p was overexpressed in the circulating exosome of diabetic patients and impaired diabetic wound healing via targeting VEGFA and inhibiting the function of HSFs 86. Circulating exosomes of diabetic patients inhibited angiogenesis in diabetic wound healing via transferring miR-15a-3p and inhibiting NOX5/ROS signaling pathway 87. Circulating exosomal miR-20b-5p inhibited angiogenesis in diabetic wound healing via inhibiting the Wnt9b/β-catenin pathway 88. Circulating exosomal miR-181b-5p promoted cell senescence and inhibited angiogenesis to impair diabetic foot ulcer (DFU) healing via the NRF2/HO-1 pathway 89.

#### 4.1.2 Exosomes in diabetic wound healing application

MSC-exos have emerged as an attractive approach in diabetic wound regeneration due to their excellent pro-regenerative and immunoregulatory functions. Exosomes from ADSCs, hucMSCs, BMSCs, induced pluripotent stem cells (iPSCs), menstrual blood-derived MSCs, and human urine-derived stem cells were proven to accelerate the healing of diabetic wounds. The current evidence on exosomes for the treatment of diabetes is largely based on the results of animal experiments and in vitro experiments. ADSC-exos protected the skin cells against oxidative stress damage and enhanced angiogenesis through activating the eHSP90/LRP1/AKT axis and enhancing SIRT3 and SOD2 expression, ultimately promoting diabetic wound healing 90 91. Diabetic ADSC-exos promoted diabetic wound healing via stimulating macrophages to secrete more TGF-β1 to activate the TGF-β/Smad3 signaling pathway in fibroblasts 92. HucMSC-exos reduced oxidative stress, and enhanced collagen deposition, angiogenesis, and M2 macrophage polarization to accelerate diabetic wound healing 93 94. Exosomes from menstrual blood-derived mesenchymal stem cells (MenSC-exos) promoted angiogenesis and M2 macrophage polarization and reduced scar formation in diabetic wound healing, and was mainly mediated by NF-κB pathway 95. Exosomes from human urine-derived stem cells were reported to promote angiogenesis in diabetic wounds via transferring DMBT1 96.

The ncRNAs, including miRNAs, lncRNAs, and circRNAs are crucial bioactive substances in MSC-exos, which are widely involved in the processes of angiogenesis, collagen synthesis, inflammation regulation, and ECM remodeling. HucMSC-exos improved angiogenesis in the diabetic wound by transferring lncRNAs related to angiogenesis, such as PANTR1, H19, OIP5-AS1, and NR2F1-AS1 97. Hair follicle MSC exosomal lncRNA H19 promoted proliferation and migration and inhibited NLRP3-related pyroptosis in keratinocytes to promote diabetic wound healing 98. In addition, genetic engineering editing in MSCs can improve the therapeutic effect of MSC-exos on diabetic wounds. Exosomes secreted by multiple stem cells play an active role in repairing diabetic wounds, including miR-132-overexpressing ADSCs 99, linc00511-overexpressing ADSCs 100, lncRNA KLF3-AS1-overexpressing BMSCs 101, HOTAIR-overexpressing BMSCs 102, lncRNA H19-overexpressing BMSCs 103, mmu_circ_0000250-overexpressing ADSCs 104, mmu_circ_0001052-overexpressing ADSCs 105, circ-Astn1-overexpressing ADSCs 106, circHIPK3-overexpressing hucMSCs 107, circ-ITCH-overexpressing BMSCs 108, and Nrf2-overexpressing ADSCs 109. The detailed information can be seen in **Table 3**.

In addition to genetic engineering editing, drug pretreating, and hypoxic culture can also improve the therapeutic effect of MSC-exos. Exosomes from melatonin-stimulated MSCs improved diabetic wound healing and promoted macrophage M2 polarization via inhibiting PTEN/AKT pathway 110. Exosomes from MSCs pretreated with pioglitazone (PGZ-Exos) improved angiogenesis in diabetic wound healing via activating the PI3K/AKT/eNOS pathway 111. Exosomes from atorvastatin-pretreated MSCs improved angiogenesis via delivering miR-221-3p and activating AKT/eNOS pathway 112.

Exosomes from hypoxia ADSCs improved the function of fibroblasts, including proliferation, migration, and collagen formation in diabetic wound healing via activating PI3K/Akt pathways 113.

Exosomes from blood can also be used to treat diabetic wounds. Exosomes derived from platelet-rich plasma (PRP) promoted the re-epithelization and angiogenesis in diabetic wounds via activating RhoA/YAP signaling 114. PRP-exos containing sphingosine-1-phosphate (S1P) promoted angiogenesis in diabetic wound healing via the S1PR1/AKT/FN1 pathway 115. PRP-exos inhibited apoptosis and pyroptosis of DFU fibroblasts to accelerate DFU healing via lncRNA MALAT1 /miR-374a-5p/ DNMT3A 116. Serum exosomes from normal healthy mice accelerated diabetic wound healing by enhancing angiogenesis and collagen synthesis 117.

In summary, MSC-exos can regulate diabetic wound healing via multiple mechanisms, including promoting proliferation and angiogenesis, reducing inflammatory response, and combating oxidative stress. Multitude origins of MSC-exos uniformly contribute to the facilitation of wound repair, potentially via distinct underlying mechanisms. Pomatto et al. compared the differential therapeutic effects of BMSC-exos and ADSC-exos on diabetic ulcer wound healing, confirming that BMSC-exos possessed a major effect on cell proliferation, while ADSC-exos were mainly involved in the angiogenesis process 118. iPSC-exos presented similar vascularization bioactivity compared with iPSC-derived MSC-exos, but with superior anti-inflammatory effect 119. In addition, genetic engineering modification and drug preconditioning can improve the efficacy of MSC-exos in the treatment of diabetic wounds.

### 4.2 Hypertrophic scar and keloid

Hypertrophic scars and keloids are abnormal wound-healing responses characterized by excessive collagen production and fibrosis 120. The pathogenesis of hypertrophic scars and keloids is believed to involve a combination of genetic, mechanical tension, and growth factor dysfunction. Exosome-mediated cellular communication between fibroblasts and immune cells participates in the formation of hypertrophic scar and keloid 121 **(Figure 2B)**.

#### 4.2.1 Exosomes in the pathogenesis of hypertrophic scar and keloid

Exosomes derived from hypertrophic scar fibroblasts have been found to play a significant role in the development of hypertrophic scars. Cui et al. found that exosomes from human hypertrophic scar fibroblasts (HHSFs) induced the EMT, and increased the expression of fibronectin, type Ⅰ and Ⅲ collagen in human normal fibroblasts (HNFs) via activating SMAD and TAK1 signaling 122. This suggested that HSF-derived exosomes can modulate the behavior of normal fibroblasts and promote the production of ECM components. In another research, HHSFs-derived exosomes promoted the proliferation, migration, and EMT and differentiation marker KRT1 and KRT10 expression of normal human keratinocytes (NHK) 123. In the context of keloids, miR-21 expression was significantly higher in exosomes from keloid fibroblasts (KFs) compared to that in normal fibroblasts 124. Exosomal miR-21 promoted the collagen production and proliferation of KFs via targeting Smad7 and activating the TGF-β/Smad pathway. This suggested that miR-21-containing exosomes derived from KFs may contribute to the pathogenesis of keloids.

M2 macrophages play a crucial role in scar formation and tissue remodeling via promoting the differentiation of fibroblasts into myofibroblasts and collagen deposition. Chen et al. found that M2 macrophage exosomes increased the expression of fibronectin, collagen I and III, and α-SMA in HDFs by transferring lncRNA-ASLNCS5088 125. Exosomal lncRNA-ASLNCS5088 promoted HDF fibrosis by targeting miR-200c-3p and increasing glutaminases (GLAs) expression. In mice, the exosome inhibitor GW4869 reduced GLS, α-SMA, and collagen synthesis after wound healing. Therefore, the inhibition of M2 macrophage exosomal lncRNA-ASLNCS5088 might be a novel therapeutic target for hypertrophic scar therapy. In addition, LINC01605 from M2 macrophage exosomes promoted the initiation and formation of hypertrophic scars via targeting miR-493-3p and activating AKT pathway 126. Hu et al. found that the has-circ-0020792 level was increased and the miR-193a-5p level was reduced in plasma exosomes in keloid patients. Simultaneously, exosomal has-circ-0020792 promoted proliferation, migration, and ECM synthesis of HNFs via inhibiting miR-193a-5p and activated TGF-β1/Smad signaling 127. Furthermore, melanocytes have been implicated in keloid formation and melanocytes in keloids exhibited increased activity compared to those in normal scars. Shen et al. found that melanocyte-derived exosomes activated the TGF-β/Smad pathway and ECM synthesis in fibroblasts, thereby contributing to the occurrence and development of keloids 128. Specifically, melanocyte-derived exosomal miR-7704 was shown to promote collagen synthesis and activate the TGF-β/Smad pathway via targeting Smurf1. Hence, exosome-mediated communication between melanocytes and fibroblasts contributed to the excessive ECM synthesis and scar formation.

#### 4.2.2 Exosomes in the treatment of hypertrophic scar and keloid

ADSC-exos enrich in anti-fibrotic cargos and represent promising effects in inhibiting fibroblast activity and collagen deposition, as well as reducing scar formation. ADSC-exos inhibited the proliferation, migration, and collagen synthesis, and promoted the apoptosis of KFs by inhibiting TGF-β1/Smad pathway 129. For instance, ADSC-exos inhibited the bioactivity, collagen deposition, and myofibroblast trans-differentiation of HHSFs via transferring miR-192-5p and inhibiting IL-17RA/SMAD pathway, and ADSC-exos also decreased collagen deposition during wound healing 130. Thus, ADSC-exos had the potential to modulate fibroblast behavior and reduce scar formation. MiR-29a, another miRNA of interest, has been found to be down-regulated in scar tissues and HHSFs. MiR-29a-overexpressing hADSC-exos inhibited the proliferation, migration, and collagen deposition of HHSFs, and reduced scar hyperplasia after scalding of mice via inhibiting TGF-β2/Smad3 signaling pathway 131. Furthermore, hADSC-exos were found to suppress the expression of ECM-related proteins in KFs, reduced collagen production, and angiogenesis in keloid tissue explants via inhibiting TGF-β2/Smad3 and Notch-1 signaling 132. Additionally, hADSC-exos-derived miR-7846-3p reduced proliferation and pro-angiogenic effects of KFs via targeting NRP2 and inhibiting the Hh signaling 133.

### 4.3 Skin aging

Skin aging is a natural process characterized by the gradual loss of skin elasticity, and firmness, and the appearance of wrinkles and age spots 134. Sun exposure, pollution, smoking, and poor skincare habits can accelerate the aging process. These factors lead to oxidative stress, inflammation, and the breakdown of collagen and elastin fibers in the skin 135. Stem cells and their paracrine products are proven to restore fibroblast function and reverse skin aging 136 **(Figure 2C)**.

#### 4.3.1 Exosomes in combating skin aging

Recently, exosome-mediated cell communication was shown to restore the senility of fibroblasts and remodel ECM in the skin. HUVEC-exos significantly increased cell proliferation and collagen type Ⅰ expression while decreasing matrix metalloproteinases-1 (MMP-1) expression of UVB-treated fibroblasts 137. Trophoblast-derived exosomes restored the senility of replicative senescent HDFs and UVB-treated HDFs via up-regulating CXCL family gene expression 138. Pretreatment with iPSC-exos reduced the expression level of senescence marker β-galactosidase (SA-β-Gal), MMP-1, and MMP-3, and restored the expression level of collagen type I in UVB-induced photo-aged HDFs and replicative senescence HDFs 139. UVB radiation activated the MAPK/AP-1 pathway in the skin, leading to an increased production of MMPs. These MMPs, in turn, degrade collagen and elastin proteins in the skin. BMSC-exos were reported to protect our skin from UVB-induced skin aging via inhibiting MAPK/AP-1 activation 140. Besides, ADSC-exos also ameliorated the aging phenotype of photo-aged HDFs 141. Mechanically, ADSC-exos reversed UVB-induced DNA damage and ROS production in fibroblasts by activating the Nrf2 pathway, reduced MMP-1 expression via inhibiting MAPK/AP-1 pathway, and promoted collagen synthesis via activating the TGF-β/Smad pathway.

Exosomal miRNAs function as a communication medium, playing a significant role in the exosome-mediated mechanisms underlying anti-aging effects. BMSC-exos promoted collagen production and reduced oxidative stress accumulation in UVB-irradiated HDFs via transferring miR-29b-3p 142. In addition, exosomes from embryonic stem cells were reported to inhibit replicative, adriamycin-induced, and ionizing radiation-induced HDF senescence via transferring miR-291a-3p and inhibiting TGFBR2 expression 143. Gao et al. found that miR-1246-overexpressing ADSC-exos exhibited an excellent improvement effect on wrinkle formation, epidermis thickening, and collagen fiber reduction of the UVB-induced photo-aging mouse model 144. The beneficial effects of miR-1246-overexpressing ADSC-exos were realized by inhibiting the MAPK/AP-1 and NFκB signaling pathways and activating TGF-β/SMAD signaling pathways.

Moreover, exosomal proteins also contributed to the anti-aging effects of exosomes. Wu et al. reported that hucMSC-exos reduced expression of aging makers p-p65, proliferating cell nuclear antigen (PCNA), and DNA damage markers in UV radiation-induced skin models via delivering natural protein 14-3-3ζ. Besides, exosomal 14-3-3ζ protects skin keratinocytes from oxidative stress via up-regulating SIRT1 expression and activating autophagy 145. ANP32B expression was significantly lower in the old skin compared to young skin, and dermal stem/progenitor cell (DSPC) exosomal ANP32B improved collagen synthesis of fibroblasts via activating Akt phosphorylation 146. Recently clinical trials also confirmed the clinical application prospect of ADSC-exos in improving skin aging. A randomized split-face study showed that the side treated with hADSC-exos and microneedling exhibited significant clinical improvements in skin wrinkles, elasticity, hydration, and pigmentation, compared to the control side treated with normal saline solution and microneedling 147.

3D spheroid culture strategy is proven to enhance the production of exosomes and improve cellular regenerative function by enhancing the paracrine effects of parental cells. Exosomes from the 3D culture of HDFs (3D-HDF-exos) increased collagen biosynthesis and ameliorated inflammation in the UVB radiation-treated skin via activating TGF-β and inhibiting TNF-α pathway 148. Besides, 3D-HDF-exos exhibited higher efficacy in reversing wrinkles than 2D-HDF-exos and MSC-exos in a nude photoaging mouse model. Exosomes from hucMSCs grown in a 3D culture system (3D-hucMSC-exos) enhanced the proliferation and migration of normal HaCaT cells 149. And 3D-hucMSC-exos increased the collagen type I expression and reduced MMP1 expression of UVB-induced photo-aged HaCaT cells. In conclusion, exosomes represent a promising cell-free therapeutic approach with potential implications for the treatment of skin aging.

## 5. Clinical application of exosomes in dermatology

Exosomes have gained significant attention in recent years. Exosomes have prompted exploration as promising therapeutic agents, diagnostic biomarkers, and potential targets for personalized medicine in dermatology.

For exosomes as therapeutic agents, the current clinical research on exosome-related therapy is still in the early stages. Currently, exosomes are clinically effective in promoting wound healing, resisting skin aging, and treating scars, but research on treating inflammatory skin diseases and autoimmune skin diseases is still in the preclinical stage. In a clinical trial, Johnson et al. suggested that human platelet exosome could be safely administered for wound healing and there were no adverse reactions 150. For skin aging, Park et al. performed prospective, randomized, split-face study, thus confirming that the side treated with hADSC-exos and microneedling exhibited significant clinical improvements in skin wrinkles, elasticity, hydration, and pigmentation, compared to the control side treated with normal saline solution and microneedling 147. Another clinical study confirmed that combination treatment with the ADSC-exos and fractional CO2 laser can significantly improve acne scars compared to only laser therapy 151.

In the aspect of diagnosis application, miRNA and cytokines in exosomes have been shown to assist in the diagnosis of autoimmune diseases and inflammatory skin conditions. In psoriasis, the circulating exosomal IL-23 and TNF-α mRNA in psoriasis patients were significantly higher than those in healthy controls, and the level of circulating exosome IL-23 was significantly positively correlated with the severity index score 24. In SLE, circulating exosome miR-21 and miR-155 in SLE patients were significantly higher than those in healthy controls, and the expression levels were even higher in SLE patients with LN 53.

Although many studies have demonstrated that exosomes are crucial in the pathogenesis of skin diseases, there are currently no clinical studies on exosomes as personalized therapeutic targets for skin diseases.

Exosomes facilitate critical intercellular communication between cutaneous cells and immune cells; consequently, future endeavors might focus on attenuating disease progression in dermatological disorders by impeding exosomal secretion. In addition, under pathological conditions, the contents of exosomes secreted by skin cells or immune cells have changed. Future studies can intervene and treat key aspects of skin diseases by regulating exosome components.

## 6. Conclusions and expectations

Exosomes are pivotal paracrine mediators in the microenvironment of skin tissue and are involved in the pathophysiological processes of many skin diseases. Here, we reviewed the value of exosomes in the pathogenesis, diagnosis, and treatment of skin diseases, with emphasis on the role of endogenous exosomes in triggering skin diseases and the role of exosomes derived from exogenous stem cells in skin tissue repair.

The mechanisms and clinical applications of exosomes differ in inflammatory skin diseases, autoimmune skin diseases, and skin regeneration. In inflammatory skin diseases, exosomes are mainly involved in the communication between keratinocytes and immune cells. Exosome-related therapy primarily functions by suppressing inflammation and promoting tissue repair to alleviate inflammation and lesions. The bioactive molecules within exosomes can inhibit the production of inflammatory mediators and activation of inflammatory cells, while promoting tissue regeneration and regulating the skin microenvironment. In autoimmune skin diseases, circulating exosomes play an important role in pathogenesis. Exosomes may alleviate inflammation and autoimmune responses by modulating the immune response. In skin regeneration, exosomes can mediate skin regeneration and repair. Exosomes promote cell proliferation, angiogenesis, and tissue regeneration by transporting growth factors, cytokines, and other bioactive molecules.

However, despite the promising findings, there still are some challenges that are worthy of attention. Firstly, there are limitations of exosome isolation and purification. The current methods for isolating exosomes, such as ultracentrifugation and size exclusion chromatography, are time-consuming, labor-intensive, and may result in low yields 152. Moreover, the isolated exosomes are usually accompanied by other extracellular vesicles or contaminants. Therefore, it is needed to seek for novel extraction methods to improve the production and concentration of exosomes. Secondly, exosomes are a heterogeneous group of cell-derived membranous structures 153. Exosomes derived from different cell types or under different physiological or pathological conditions can change their cargo composition and functional properties. The heterogeneity of exosomes poses challenges in deciphering the specific roles of exosomes in skin diseases and developing targeted therapies. Due to the heterogeneity of exosomes and the complexity of the skin microenvironment, it is difficult to repeat the therapeutic effect of exosome therapy in clinical practice. Further research is needed to characterize the molecular and functional diversity of exosomes and identify specific markers or signatures that can be used to distinguish subpopulations with distinct functions. Thirdly, exosomes are vesicles carrying multi-component cargoes. The current application of exosomes in the diagnosis of skin diseases mainly depends on one specific miRNA or protein 154. It is necessary to construct signatures based on multiple disease-specific biomarkers in exosomes for the diagnosis and severity grading of skin diseases. In addition, the current research on exosomes in the mechanism and treatment of dermatosis mainly focuses on cellular and animal levels. But human skin has significant species differences from animal skin. For example, mouse skin is thinner and looser than human skin, and hair follicles in mice are more abundant than in humans 155. Therefore, the results of preclinical studies cannot fully reflect and predict the functional mechanisms and therapeutic effects of exosomes in human skin disease. Clinical trials or animal models closer to human skin to verify the role of exosomes in skin diseases are urgent. Finally, although engineered exosomes and drug-preconditioning exosomes have been extensively investigated in the realm of tissue regeneration and tumors, they have rarely been studied in inflammatory and autoimmune skin diseases. Future studies could consider performing exosome modification to improve the efficacy of exosomes in curing skin diseases.

In light of these limitations above, these directions need to be paid attention to in future research. Efficient, stable, and mass-producible exosomes for the treatment of skin disease are yet to be explored and developed. Fully studying the heterogeneity of skin diseases and the diversity of exosome containings can explore novel therapeutic targets for skin diseases and improve the reproducibility of exosome-related therapy. At present, exosome-related therapy is still mainly in the pre-clinical stage, and the therapeutic effect needs to be proved by multi-center clinical studies. In addition, physical, chemical, or biological methods for preconditioning, genetic engineering, and transfection were proven to enhance the therapeutic potential of exosomes It is a new therapeutic direction to explore the engineering strategy of modifying exosomes to improve the efficacy of exosomes in the treatment of skin diseases.

In conclusion, our review provided novel insight into the involvement of exosomes in the pathological process, diagnosis, and treatment of skin disease. Further studies are needed to explore deeper mechanisms related to exosomes in dermatosis progress and to optimize the function of exosomes for clinical treatment of dermatosis.

## Figures and Tables

**Figure A:**
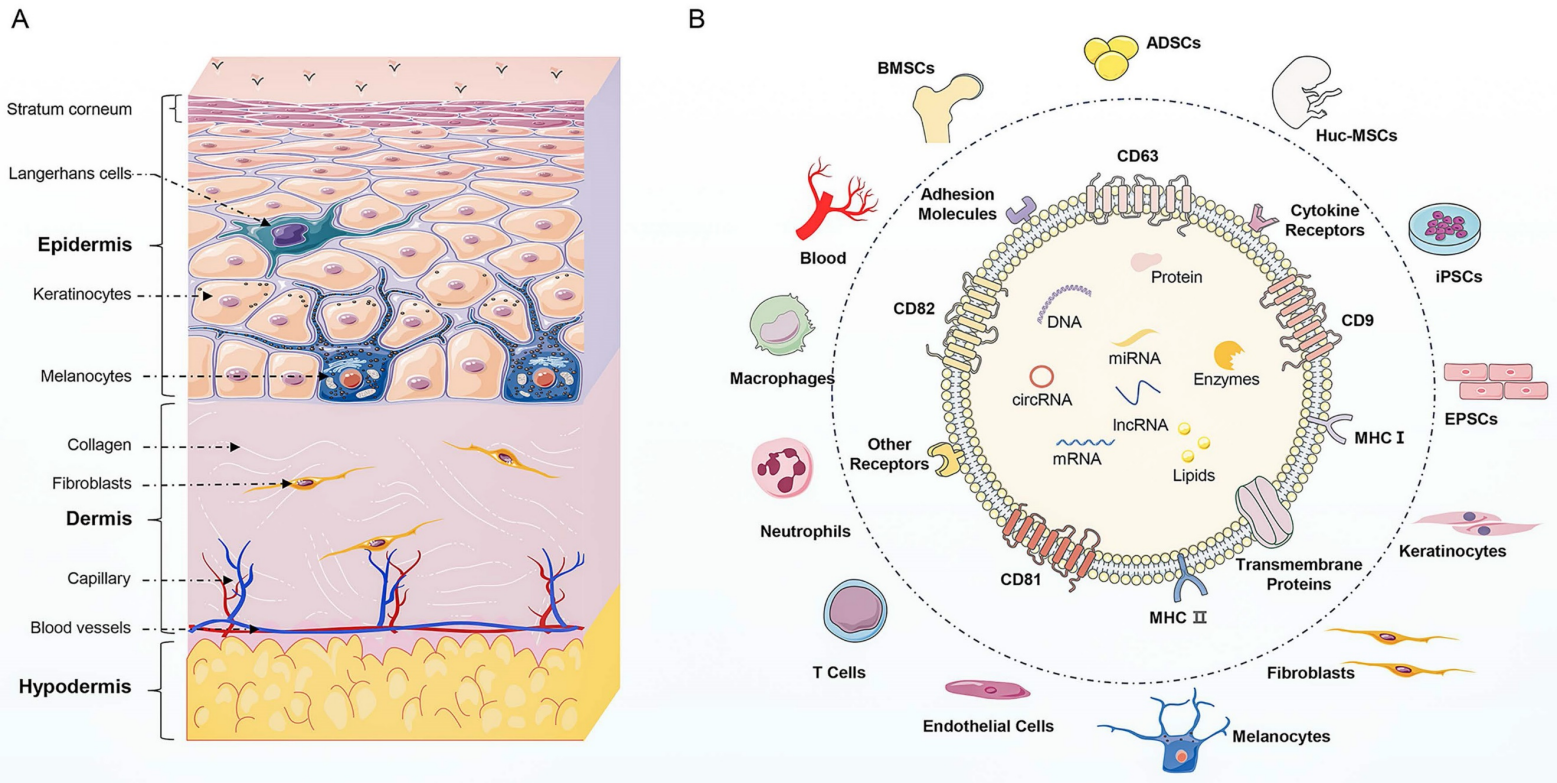
** (Summary Figure). The structural organization of the skin and the compositions of exosomes.** (A) Skin is composed of epidermis, dermis, and hypodermis. The epidermis consists of keratinocytes, along with melanocytes and Langerhans cells. The dermis contains fibroblasts, which produce collagen and elastin fibers comprising extracellular matrix, that provide strength and elasticity to the skin. The dermis also contains blood vessels and nerve endings. The hypodermis is composed of adipocytes, mesenchymal stem cells, and blood vessels. (B) The source of exosomes involved in the regulation of skin function mainly include skin cells (keratinocytes, fibroblasts, melanocytes, and endothelial cells), immune cells (T cells, neutrophils, and macrophages), stem cells (ADSCs, BMSCs, hucMSCs, iPSCs, and EPSCs) and blood. Besides, exosomes display similar biomarkers, including tetraspanins (CD81, CD82, CD9), heat shock proteins (HSP70, HSP90), biogenesis-related proteins (ALIX, TSG101), and major histocompatibility complex (MHC-I and MHC-II). Additionally, exosomes enrich in multiple bioactive cargoes, mainly proteins, lipids, and nucleic acid, including DNA, mRNA, miRNA, lncRNA, and circRNA.

**Table 1:** The mechanisms of exosomes in vitiligo pathogenesis

Exosome Source	Model	Mechanism	Ref.
Blood of segmental vitiligo patients	Human primary keratinocytes and melanocytes	MiR-493-3p-overexpressing keratinocyte induced melanocyte apoptosis and decreased melanin synthesis through miR-493-3p/HNRNPU/COMT/dopamine axis	(46)
Peripheral blood of vitiligo patients	Human melanocytes cell	Circulating exosomes inhibited melanin content, tyrosinase activity, and melanogenesis-related protein expression via transferring miR-21-5p and inhibiting SATB1 expression	(47)
Serum of progressive vitiligo patients	Progressive vitiligo patients	Hsa-miR-487b-3p was significantly lower and positively correlated with the area of lesions	(14)
Keratinocytes in vitiligo lesions	NHEM	MiR‐200c up-regulated the expression of melanogenesis‐related genes via inhibiting SOX1 and activating β‐catenin	(48)

**Table 2:** The mechanisms of exosomes in systemic lupus erythematosus pathogenesis

Exosome Source	Model	Mechanism	Ref.
Serum of SLE patients	PBMCs	Up-regulated the expression of IFN-α, TNF-α, IL-1β, and IL-6 in healthy peripheral blood mononuclear cells (PBMCs) in a TLR-dependent manner	(51)
Plasma of SLE patients		Exosome-delivered microRNAs promote IFN-α secretion by human plasmacytoid DCs via TLR7	(54)
Blood of SLE patients	SLE patients	Differently expressed exosomal miRNAs and proteins mainly enriched in pathways associated with inflammation and autophagy	(52)
Serum of SLE patients with or without lupus nephritis		MiR-21 and miR-155 were elevated in the serum of SLE patients compared to healthy controls, with even higher levels in SLE patients with LN	(53)
Serum of SLE patients		MiRNAs containing an IFN induction motif promoted activation, maturation, and survival of human plasmacytoid DCs via stimulating TLR7 expression	(55)
T cells of Lck-BPI Tg mice	C57BL/6J mice	Increased autoantibody levels, tissue IFN-γ levels, and induced hepatitis, nephritis, and arthritis in recipient mice	(56)
T cells of T-cell-specific BPI transgenic mice		Stimulated IL-1β production of macrophages and induced liver, kidney, and joint inflammatory responses	(57)

**Table 3:** The mechanism of gene-modified MSC-exos in diabetic wound healing

Gene Modification	Exosome Source	Model	Mechanism	Ref.
MiR-132 overexpressing	ADSCs	Backside full-thickness skin wounds and random skin flaps in STZ-induced diabetic mice	Promoted angiogenesis and M2-macrophages polarization via inhibiting NF-κB signaling pathway	(99)
Linc00511 overexpressing		Rat DFU models	Accelerated angiogenesis via up-regulating PAQR3 expression and suppressing Twist1 ubiquitin degradation	(100)
Mmu_circ_0000250 overexpressing		Backside full-thickness skin wounds in STZ-induced diabetic mice	Activated autophagy and promoted angiogenesis of EPCs via inhibiting miR-128-3p to up-regulating SIRT1 axis	(104)
Mmu_circ_0001052 overexpressing		Mouse DFU models	Promoted angiogenesis via miR-106a-5p/FGF4/p38MAPK axis	(105)
Circ-Astn1 overexpressing		Backside full-thickness skin wounds in STZ-induced diabetic mice	Suppressed apoptosis and improved angiogenesis via miR-138-5p/SIRT1/FOXO1 axis	(106)
Nrf2 overexpressing		Rat DFU models	Improved glucose-induced EPC senescence and inhibited ROS production and inflammatory cytokine expression	(109)
LncRNA KLF3-AS1 overexpressing	BMSCs	Backside full-thickness skin wounds in STZ-induced diabetic mice	Stimulated angiogenesis via reversing miR-383-mediated VEGFA inhibition	(101)
HOTAIR overexpressing		Backside full-thickness skin wounds in db/db mice	Enhanced angiogenesis via up-regulating VEGF	(102)
LncRNA H19 overexpressing		Mouse DFU models	Inhibited the apoptosis and inflammation of fibroblasts via sponging miR-152-3p and activating PTEN expression to reduce AKT phosphorylation	(103)
Circ-ITCH overexpressing		Mouse DFU models	Inhibited ferroptosis and improved angiogenesis by recruiting TAF15 and activating the Nrf2 signaling pathway	(108)
CircHIPK3 overexpressing	HucMSCs	Mouse DFU models	Promoted angiogenesis via inhibiting miR-20b-5p to activate Nrf2 and VEGFA expression	(107)

**Table 4:** The mechanism of exosomes in combating skin aging

Exosome Source	Model	Mechanism	Potential Application	Ref.
HUVECs	UVB-treated fibroblasts	Increased cell proliferation, collagen type Ⅰ, and decreased matrix MMP-1expression	Ameliorated the photo-aging of fibroblasts	(137)
Trophoblasts	Replicative senescent HDFs and UVB-treated HDFs	Up-regulated CXCL family gene expression	Restored the senility of fibroblasts	(138)
iPSCs		Reduced the expression of SA-β-Gal, MMP-1, and MMP-3, and restored the expression of collagen type I	Ameliorated the photo-aging of fibroblasts	(139)
BMSCs	UVB-treated HDFs	Inhibited MAPK/AP-1 pathway activation	Protect skin from aging	(140)
ADSCs		Activated Nrf2 pathway, TGF-β/Smad pathway and inhibited MAPK/AP-1 pathway	Ameliorated the aging phenotype of fibroblasts	(141)
BMSCs		Promoted collagen production and reduced oxidative stress accumulation via transferring miR-29b-3p	Ameliorated the photo-aging of fibroblasts	(142)
Embryonic stem cells	Replicative, adriamycin-induced, and ionizing radiation-induced HDFs	Transferred miR-291a-3p and inhibited TGFBR2	Ameliorated senescence of fibroblasts	(143)
MiR-1246-overexpressing ADSCs	UVB-induced photo-aging mice	Inhibited the MAPK/AP-1 , NFκB pathways and activated TGF-β/SMAD pathways	Improved wrinkle formation, and epidermis thickening.	(144)
HucMSC	UV radiation-induced SD rats' acute skin photodamage	Transferred14-3-3ζ, up-regulated SIRT1 expression, and activated autophagy	Alleviated UV radiation-induced photodamage	(145)
DSPCs	Fibroblasts	Transferred ANP32B and activated Akt phosphorylation	Improved collagen synthesis of fibroblasts	(146)
hADSCs	Human with facial skin aging	Promoted collagen synthesis and deposition	Improved skin wrinkles, elasticity, hydration, and pigmentation	(147)
3D-HDFs	UVB radiation-treated skin and nude photoaging mice	Increased collagen biosynthesis and ameliorated inflammation via activating TGF-β and inhibited TNF-α pathway	Ameliorated skin photoaging	(148)
3D-hucMSCs	UVB-induced photo-aged HaCaT cells	Enhanced the proliferation, migration, and collagen type I synthesis and reduced MMP1	Ameliorated HaCaT Cell photo-aging	(149)

## Abbreviations

ADSCs: Adipose-derived stem cells
AD: atopic dermatitis
BPI: bactericidal/permeability-increasing protein
BMSCs: bone marrow mesenchymal stem cells
CCLs: C-X-C motif chemokine ligands
DCs: dendritic cells
DSPC: dermal stem/progenitor cell
DFU: diabetic foot ulcer
dSSc: diffuse cutaneous systemic sclerosis
DNCB: dinitrochlorobenzene
EPC-exos: endothelial progenitor cell-derived exosomes
ECP: eosinophil cationic protein
ESCs: epidermal stem cells
3D-hucMSC-exo: exosomes from hucMSCs grown in a three-dimensional culture system
PGZ-Exos: exosomes from MSCs pretreated with pioglitazone
ECM: extracellular matrix
EVs: extracellular vesicles
GLAs: glutaminases
HDFs: human dermal fibroblasts
HDMECs: human dermal microvascular endothelial cells
HHSFs: human hypertrophic scar fibroblasts
HNFs: human normal fibroblasts
hucMSCs: human umbilical cord MSCs
IMQ: imiquimod
iMSC: induced pluripotent stem cell-derived mesenchymal stem cells
iPSCs: induced pluripotent stem cells
KFs: keloid fibroblasts
LN: lupus nephritis
MMP-1: matrix metalloproteinases-1
MenSC-exos: menstrual blood-derived mesenchymal stem cells
MSCs: mesenchymal stem cells
NET: neutrophil extracellular trap
NHK: normal human keratinocytes
PC: phosphatidylcholine
PE: phosphatidylethanolamine
PRP: platelet-rich plasma
PCNA: proliferating cell nuclear antigen
ROC: receiver operating characteristic
SV: segmental vitiligo
SA-β-Gal: senescence marker β-galactosidase
S1P: sphingosine-1-phosphate
SLE: systemic lupus erythematosus
SSc: systemic sclerosis
TLR2: Toll-like receptor 2
TEWL: transepidermal water loss
tRFs: transfer RNA-derived fragments
UCB-MNC: umbilical cord blood mononuclear cells
VDR: vitamin D receptor
